# Objective audiometry with DPOAEs: New findings for generation mechanisms and clinical applications

**DOI:** 10.1007/s00106-016-0267-y

**Published:** 2017-05-03

**Authors:** D. Zelle, E. Dalhoff, A. W. Gummer

**Affiliations:** Section of Physiological Acoustics and Communication, Department of Otolaryngology, University of Tübingen, Elfriede-Aulhorn-Str. 5, 72076 Tübingen, Germany

**Keywords:** Acoustic stimulation, Hearing, Cochlear amplifier, Auditory threshold, Hearing loss

## Abstract

**Background:**

Distortion product otoacoustic emissions (DPOAEs) and transient-evoked otoacoustic emissions (TEOAEs) are sound waves generated as byproducts of the cochlear amplifier. These are measurable in the auditory canal and represent an objective method for diagnosing functional disorders of the inner ear. Conventional DPOAE and TEOAE methods permit detection of hearing impairment, but with less than desirable accuracy.

**Objective:**

By accounting for DPOAE generation mechanisms, the aim is to improve the accuracy of inner-ear diagnosis.

**Methods:**

DPOAEs consist of two components, which emerge at different positions along the cochlea and which may cause artifacts due to mutual interference. Here, the two components are separated in the time domain using short stimulus pulses. Optimized stimulus levels facilitate the acquisition of DPOAEs with maximum amplitudes. DPOAE and Békésy audiograms were recorded from 41 subjects in a clinically relevant frequency range of 1.5–6 kHz.

**Results:**

The short stimulus pulses allowed artifact-free measurement of DPOAEs. Semilogarithmic input–output functions yielded estimated distortion product thresholds, which were significantly correlated with the subjectively acquired Békésy thresholds. In addition, they allowed detection of hearing impairment from 20 dB HL, with 95% sensitivity and only a 5% false-positive rate. This accuracy was achieved with a measurement time of about 1–2 min per frequency.

**Conclusion:**

Compared to conventional DPOAE and TEOAE methods, separation of DPOAE components using short-pulse DPOAEs in combination with optimized stimulus parameters considerably enhances the accuracy of DPOAEs for diagnosing impairment of the cochlear amplifier.

Distortion product otoacoustic emissions (DPOAEs) evolve from nonlinear sound processing within the cochlea and offer noninvasive, objective diagnosis of inner-ear malfunction. Conventional DPOAE methods and transient-evoked otoacoustic emissions (TEOAEs) permit detection of hearing loss, but with less than desirable accuracy. Using short-pulse DPOAEs and data analysis accounting for DPOAE generation mechanisms, the accuracy increases considerably and a quantitative diagnosis is afforded.

Otoacoustic emissions (OAEs) are sound waves measurable in the ear canal that are triggered by an acoustic stimulus and emerge as byproducts of the nonlinear amplification process in the cochlea (Fig. 1). This process locally amplifies hydrodynamic oscillations within the cochlea, enabling excitation of the inner hair cells (IHCs) at low-to-moderate sound levels. The intact cochlear amplifier is indispensable for the low hearing threshold, high-frequency selectivity, and wide dynamic range of the human auditory system [1]. Since the discovery of OAEs by Kemp in 1978 [13], two different types of emissions have become established in the clinic for the qualitative assessment of inner-ear malfunction resulting from an impaired cochlear amplifier – transient-evoked OAEs (TEOAEs) and distortion-product OAEs (DPOAEs) [10]. TEOAEs are generated by a brief click stimulus and, owing to the broadband nature of the stimulus, present the functional integrity of the cochlear amplifier in a frequency range between about 1 and 5 kHz [11]. Assessment of the TEOAEs in a frequency range around 2 kHz has been shown to be clinically most suitable for detecting hearing loss [18].

**Fig. 1:**
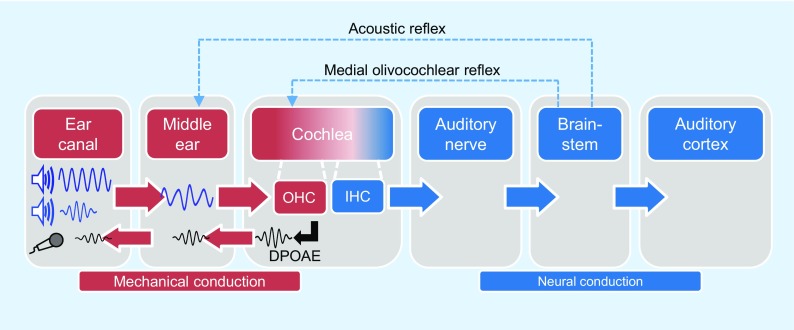
DPOAE acquisition for direct assessment of the functional integrity of the cochlear amplifier in the human auditory system. *DPOAE* distortion-product otoacoustic emissions, *IHC* inner hair cells,* OHC* outer hair cells

DPOAEs are generated by two simultaneously presented stimulus (primary) tones with frequencies f_1_ and f_2_ [14]. Suitable stimulus parameters (f_2_/f_1_ = 1.2 and L_1_ ≥ L_2_) lead to overlap of the two traveling waves induced by the primary tones in a region basal to the characteristic place of the f_2_ frequency (Fig. 2d). In this overlap region, the nonlinear dependence of the channel opening probability on deflection of the outer hair cell (OHC) stereocilia yields intermodulation between the receptor currents associated with f_1_ and f_2_. In other words, intermodulation products in the receptor current result from the nonlinear form of the transfer function of the mechanoelectrical transducer (MET). The electromechanical transducer (EMT) of the OHC soma transduces these intermodulation receptor voltages into forces, which are then coupled into the cochlear partition and fluids. Part of the resulting vibrations propagate retrogradely toward the cochlear base, drive the middle ear, and are measurable in the ear canal as DPOAEs [1].

In humans, the most pronounced distortion product emerges at the cubic difference frequency f_DP_ = 2f_1_-f_2_; it offers *frequency-specific* information about the functional integrity of the cochlear amplifier. Typically, the DPOAE amplitude or level is displayed as a function of stimulus frequency; the plot is called a DP-gram.

**Fig. 2:**
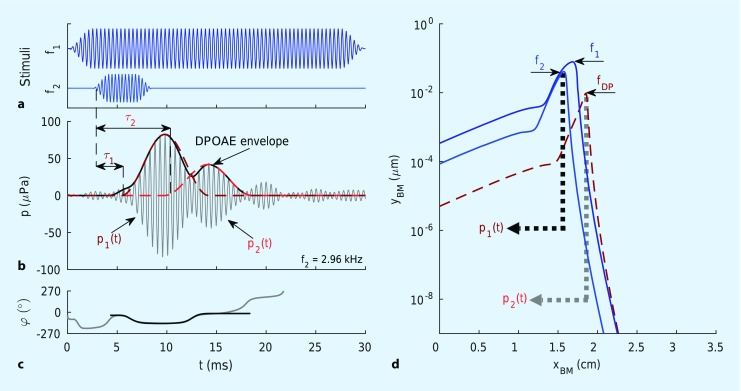
Short-pulse stimulation of the cochlea for separating DPOAE components into time and phase signals. **a** The two stimulus tones at frequencies f_1_ (30 ms pulsed, “quasi-continuous”) and f_2_ (“short-pulsed”). **b** Time signals. *Gray line* measured time signal. *Dark red dashed line* envelope of the calculated nonlinear distortion component, p_1_(t). *Light red dashed line* envelope of the calculated coherent reflection component, p_2_(t). *Black line* envelope of the calculated DPOAE signal, p_1_(t) + p_2_(t). Relative to the start of the f_2_-pulse, p_1_(t) is delayed by τ_1_ and p_2_(t) by τ_2_. **c** Phase signals. *Gray line* measured phase signal. *Black line* phase signal of the calculated DPOAE signal, p_1_(t) + p_2_(t). **d** Generation of the DPOAE source components. Envelopes of the traveling waves of the tones as function of distance from the basal end of the basilar membrane (*BM*), x_BM_. *Dark blue line*: f_2_-tone. *Light blue line*: f_1_-tone. *Red dashed line* f_DP_-tone. p_1_(t) is generated near the maximum of the traveling-wave envelope of the f_2_-tone and p_2_(t) near the envelope maximum of the f_DP_-tone. *DPOAE* distortion-product otoacoustic emissions

DP-grams and TEOAEs are used in clinical routine [11]. However, neither provides a truly reliable diagnosis because of the different OAE level dependencies and the variable middle-ear transfer functions across subjects.

TEOAEs can be detected up to a hearing loss of approximately 30 dB, but provide only limited information about the frequency specificity of a possible hearing loss [10]. By contrast, DPOAEs may exist even in the case of severe hearing impairment, hampering their interpretation when recorded with conventional methods. For example, DPOAEs are still detectable 2 h *post mortem* in gerbil at high stimulus levels (L_1_ = L_2_ = 70 dB SPL) [19]. This, perhaps initially puzzling, observation can be explained by the electrical energy supply of the EMT being comprised of the OHC resting membrane potential and the endocochlear potential. While the latter requires an active Na-K pump and becomes 0 mV within 2 min *post mortem*, the resting membrane potential persists *post mortem* for a much longer time owing to intra-/extracellular concentration gradients. Collapse of the endocochlear potential results in considerable decrease in cochlear amplification. However, the biomechanical structure responsible for generation of intermodulation products continues to operate and, for excitation with sufficiently high sound levels, can lead to the generation of DPOAEs. Under conventional stimulus and measurement conditions, DPOAEs are detectable in humans for hearing losses of up to 50 dB [10]. Hence, in clinical practice, it is advisable to limit DPOAEs to low-to-moderate primary-tone levels and also to account for the general level dependence of emissions [25].

## DPOAE generation in the cochlea

In general, DPOAEs consist of two components, which arise from different mechanisms at different places along the basilar membrane (BM) [20]. Close to the tonotopic place of the f_2_ tone, x_BM_(f_2_), the so-called nonlinear distortion component, p_1_(t), emerges from the nonlinear interaction of the traveling waves associated with the two primary tones (dark blue and blue lines in Fig. 2d). This component serves as an intracochlear pressure stimulus of frequency f_DP_. Thereby, a second anterograde traveling wave is generated (red dashed line in Fig. 2d), which has its maximum at the tonotopic place of the f_DP_ tone, x_BM_(f_DP_). There, a second DPOAE component, p_2_(t), called the coherent reflection component, arises as a result of coherent reflection at irregularities in mechanical impedances in the organ of Corti. Local amplification of the wave near x_BM_(f_DP_) enables coherent reflection by summation of in-phase, locally distributed reflections, analogous to the generation of stimulus-frequency OAEs [1].

Both components (the nonlinear distortion component and the coherent reflection component), each of frequency f_DP_, propagate retrogradely toward the ear canal, where they are measurable as a DPOAE signal. Depending on the amplitude ratio and the relative difference in phase between the two DPOAE components, interference between the components occurs, which is apparent as quasi-periodic variation of the DPOAE amplitude in a high-resolution DP-gram [3, 17]. This variation is known as DPOAE fine structure. For example, destructive interference, which arises for a relative phase difference of 180°, can cause near-cancelation of the DPOAE signal when the DPOAE components are of similar amplitude, misleadingly implying impairment of the cochlear amplifier. For purposes of clinical diagnosis, the coherent reflection component represents a biological interference signal when using conventional DPOAE methods.

## DPOAE source separation

**Fig. 3:**
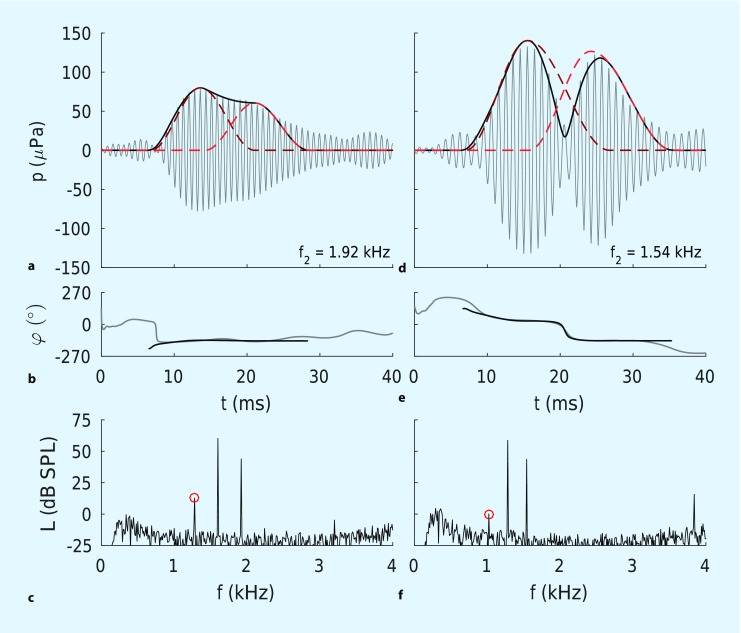
Time-domain representation of short-pulse DPOAEs for constructive (**a**, **b**) and destructive (**d**, **e**) interference, and corresponding amplitude spectra recorded with conventional continuous stimuli (**c**, **f**). *Red circles* indicate the spectral components at f_DP_ = 2f_1_-f_2_

The interference susceptibility for conventional DPOAE stimulation paradigms is due to the use of *continuous* primary tones and the extraction of the DPOAE by means of spectral analysis (Fig. 3c, f). Spectral analysis of responses for continuous, two-tone stimulation cannot distinguish between the two DPOAE components at a given distortion product frequency. Several methods have been developed to suppress or separate the coherent reflection component. For instance, Zenner and colleagues [9] suppressed the coherent reflection component using a third primary tone – called a suppressor – with a frequency similar to f_DP_. This suppression technique yields considerable reduction of fine structure. However, the optimal stimulus level of the suppressor has been shown to exhibit high variability across subjects [6]. Alternative methods require either the acquisition of a high-resolution DP-gram [12] or the use of chirp primary tones spanning a wide frequency range [16]. Both techniques provide comprehensive information beyond audiometric test frequencies, but at the expense of additional acquisition time.

Short-pulse DPOAEs in combination with time domain analysis represent a promising approach for separating the DPOAE components [22, 24], as depicted in Fig. 2. This technique exploits the different latencies of the DPOAE components [21]. By utilizing short f_2_ pulses (Fig. 2a) with length similar to the delay of the coherent reflection component relative to the nonlinear distortion component, intermodulation close to the f_2_-tonotopic place (Fig. 2d) occurs only for a limited time. The resulting nonlinear distortion component, p_1_(t), arrives at the microphone with a delay $\tau _{1}$, relative to the start of the f_2_ pulse, and decays after offset of the f_2_ pulse, while the coherent reflection component, p_2_(t), reaches the ear canal with the longer latency $\tau _{2}$ (Fig. 2b). Sampling the envelope (black line in Fig. 2b) of the short-pulse DPOAE at a time instant before the coherent reflection component starts to interfere yields the nonlinear distortion component – the relevant component for clinical assessment [22].

Another extraction technique enables the quantification of the time courses of both DPOAE components by fitting the DPOAE signal with a mathematical model [24]. Figs. 2b and 3a, d show the envelopes of the identified DPOAE components for the three interference types – quadrature, constructive, and destructive interference, respectively. Dark red dashed lines correspond to the nonlinear distortion components, p_1_(t), whereas light red dashed lines depict the coherent reflection components, p_2_(t). Figs. 2c and 3b, e depict the instantaneous phases of the measured DPOAE signal (gray line) and the computed DPOAE signal (black line) for the three types of interference. The interference type is identifiable by the time courses of the DPOAE signal and the instantaneous phase. For example, destructive interference results in a notch in the DPOAE signal in the overlap region of both components (Fig. 3d) and causes a phase jump once the coherent reflection component dominates (Fig. 3e).

## Artifact-free DPOAE acquisition

**Fig. 4:**
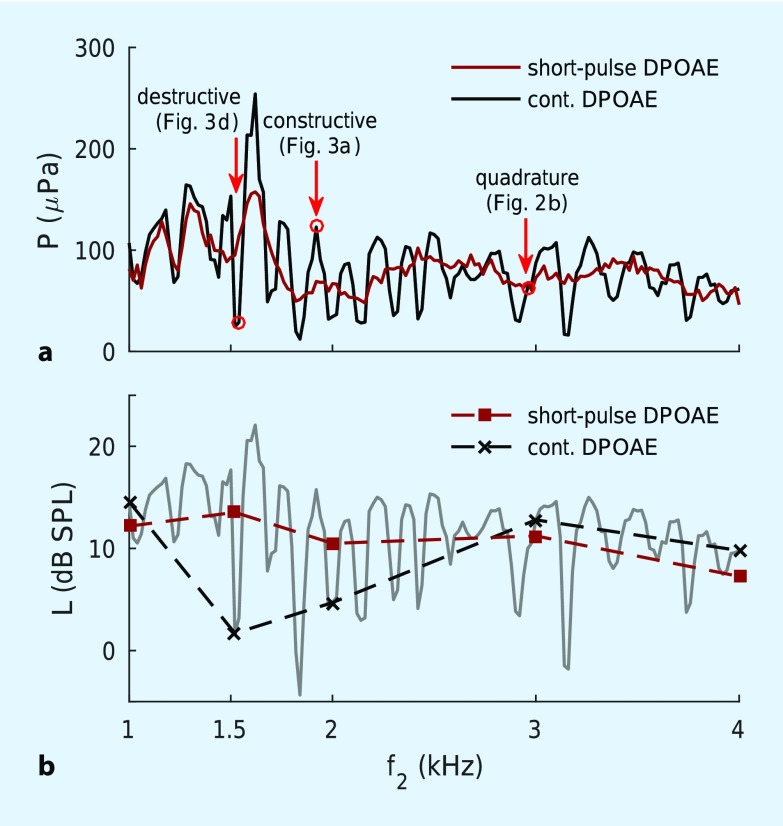
High-resolution DP-grams (**a**) and corresponding low-resolution (conventional) DP-grams (**b**) for a normal-hearing subject. *Red circles* indicate examples of frequencies (f_2_) at which destructive, constructive, and quadrature interference were observed. *DPOAE* distortion-product otoacoustic emissions

Fig. 3c, f shows amplitude spectra based on a conventional DPOAE recording for a normal-hearing subject using continuous primary tones with f_2_ = 1.92 and 1.54 kHz, respectively. The DPOAE level of 12.8 dB SPL in the case of constructive interference (Fig. 3c) exceeds the 0.2 dB SPL found for destructive interference (Fig. 3f). Fig. 4a shows the corresponding DP-gram acquired with continuous primary tones (black line). Owing to the high-frequency resolution of the DP-gram ($\Updelta$f_2_ = 20 Hz), the DPOAE fine structure becomes evident as quasi-periodic variation of DPOAE amplitude. In clinical routine, the DP-gram is usually only measured at the frequencies employed for audiograms. Such coarse frequency resolution hampers discrimination between wave-interference artifacts and frequency-specific hearing impairments. Fig. 4b shows a clinical-type DP-gram (black dashed line) reconstructed from the high-resolution DP-gram in Fig. 4a. As a result of the fine structure, DPOAE levels at f_2_ = 1.5 and 2 kHz are near 0 dB SPL, erroneously implying that the functional integrity of the cochlear amplifier is compromised at these frequency places. Short-pulse DPOAEs can reduce fine structure considerably since the DP-gram then derives solely from the nonlinear distortion component (red line in Fig. 4a). The derived DP-gram with f_2_ at the usual audiogram frequencies (red dashed line in Fig. 4b) now accurately represents the functional integrity of the cochlear amplifier at these frequency places.

## Optimization of DPOAE acquisition paradigms

The relatively poor reliability of conventional DPOAEs for identifying hearing impairments is not exclusively due to artifacts associated with wave interference between their two components. Similar to other types of OAE, DPOAE levels exhibit intersubject variability that is mainly attributed to the twofold middle-ear transmission [4], that is, to transmission through the middle ear in both the forward and reverse directions. A more advanced approach, which can circumvent middle-ear variability, employs DPOAE input–output (I/O) functions. With this technique, for every stimulus-frequency pair, the DPOAE amplitude is plotted as a function of the excitation level, L_2_, of the second primary tone. For a given stimulus-frequency pair, extrapolation to the abscissa of a linear regression line estimated from these semi-logarithmically plotted data yields an estimate of the threshold of the DPOAE, called the estimated distortion product threshold (EDPT) [2]. Ideally, the EDPT is largely independent of middle-ear transmission. This procedure requires careful selection of the pairs of primary-tone levels to ensure maximum signal amplitudes. The so-called scissor paradigm has proved successful for this purpose [15]. For a given L_2_, the value of L_1_ is chosen, independent of stimulus frequency, according to L_1_ = 0.4L_2_ + 39 dB SPL. This relation compensates for the different compressibilities of the primary-tone traveling waves at the f_2_-tonotopic place on the BM. Figure 2d illustrates the ideal case schematically, achieving identical amplitudes of the f_1_ and f_2_ traveling waves at the f_2_ tonotopic place, thus facilitating maximal overlap in the DPOAE generation region and, therefore, resulting in maximal DPOAE amplitude.

**Fig. 5:**
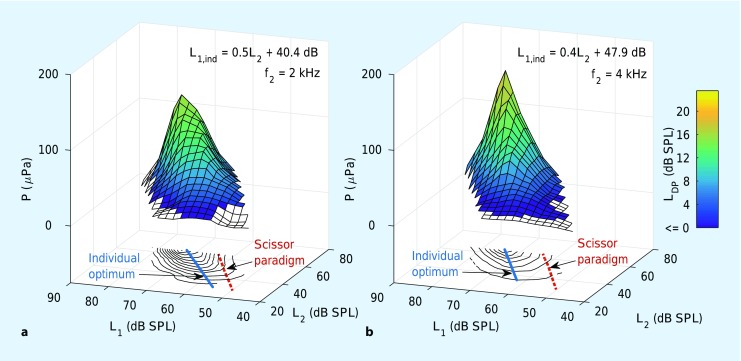
Level maps for determining optimal DPOAE stimulus levels, individually for each stimulus-frequency pair and subject. The linear equations give the optimal L_1_ value for a given L_2_ value, called the “individual optimum”, L_1,ind_

However, previous investigations attempting to define optimal stimulus levels failed to consider possible interference effects caused by the coherent reflection component. A recently published study utilized both short-pulse stimulation as well as continuous primary tones to determine optimal DPOAE stimulus levels for a clinically relevant frequency range of f_2_ = 1 to 8 kHz [25]. DPOAE level maps were used to identify optimal L_1_,L_2_ stimulus pairs for each stimulus-frequency pair and each subject. These three-dimensional maps depict DPOAE amplitude as function of L_1_ and L_2_. Figure 5a, b shows two examples of a level map from a normal-hearing subject acquired with short-pulse DPOAE at f_2_ = 2 and 4 kHz, respectively. Each L_1_,L_2_ plane depicts: (1) the contour lines of the corresponding level map (black lines), (2) the stimulus levels according to the scissor paradigm (red dashed line), and (3) the individually determined optimal L_1_,L_2_ pairs (L_1,ind_; blue line). For both level maps, it is evident that the frequency-independent scissor paradigm does not provide the optimal overlap between the traveling waves of the two primary tones. This less-than-optimal situation is apparent from the considerably smaller DPOAE amplitudes associated with the scissor paradigm compared with those from the individual, frequency-specific optimal L_1_,L_2_ pairs. The pooled data across all subjects in [25] showed significant dependence of the optimal primary-tone levels on stimulus frequency.

## Relevance for diagnostics and screening

**Fig. 6:**
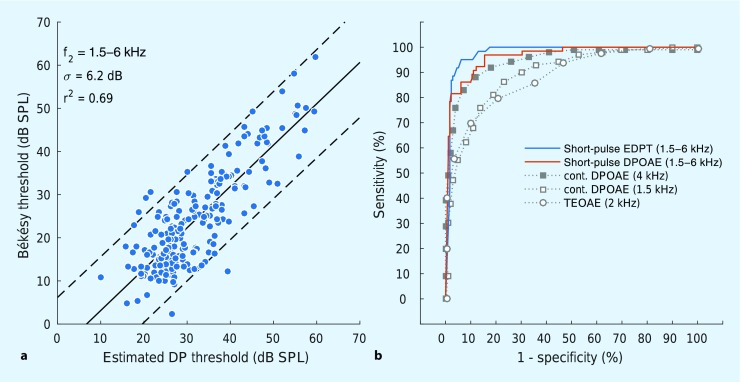
**a** Békésy threshold as a function of estimated DPOAE threshold (*EDPT*) for 41 subjects with normal hearing and sensorineural hearing loss. **b** ROC curves for the detection of hearing loss using short-pulse DPOAEs (*solid lines*) in comparison with conventional DPOAE and TEOAE methods (*dashed lines*). *DPOAE* distortion-product otoacoustic emissions, *TEOAE* transient-evoked otoacoustic emissions

Using short-pulse DPOAEs in combination with the optimal, frequency-specific primary-tone levels defined elsewhere [25] yields considerable enhancement of the accuracy of OAE diagnostics compared with hitherto existing DPOAE and TEOAE methods. From a scientific point of view, correlation between a quantitative diagnostic measure and a suitable gold standard represents the most meaningful procedure for evaluating the precision of an OAE method. In the case of DPOAEs, for example, this could be the correlation of the EDPT and the pure-tone threshold. Figure 6a depicts the correlation of EDPTs with corresponding thresholds obtained by Békésy audiometry [5, 26] for 41 subjects with normal hearing and sensorineural hearing loss, and frequencies of 1.5–6 kHz. The standard deviation of the auditory threshold estimates, σ, is 6.2 dB, the squared correlation coefficient, *r* ^2^, is 0.69, and the 95% confidence interval (dashed lines) is ±12.5 dB. The latter parameter can be interpreted as the accuracy of the diagnostic procedure. The auditory threshold estimates are approximately normally distributed: only 6/238 estimates, corresponding to 2.5% of the data, are located outside of the confidence interval; for a normal distribution one would expect 5%.

By contrast, the EDPT data gathered by Boege and Janssen [2] from subjects with normal hearing and sensorineural hearing loss using *continuous stimulation and the scissor paradigm* present larger scatter, with *r* ^2^ = 0.42 and σ = 10.9 dB. In that publication [2], the slope of the linear regression line of the auditory threshold versus the EDPT of 1.18 dB/dB suggests that losses beyond the cochlear amplifier were also present in their subjects. In the present experiments, the slope of 0.96 ± 0.04 dB/dB (Fig. 6a) implies that sensorineural hearing loss for these subjects is primarily the result of cochlear amplifier dysfunction.

For many OAE procedures, correlation analyses have not been performed or are only available to a limited extent. In practice, such analyses would not have been relevant for conventional stimulus and measurement paradigms because their inherent inaccuracy has meant that only dichotomous decisions could be made about the functional integrity of the cochlear amplifier. The statistical quality of dichotomous decisions can be evaluated with a receiver operating characteristic (ROC) curve, which plots the *sensitivity* (the true-positive rate) as function of *1-specificity* (the false-positive rate). The ROC curve associated with the aforementioned data from 41 subjects was computed for a hypothetical, dichotomous decision-making task: normal hearing – yes/no. The results can be compared with data from the literature for both conventional DPOAE and TEOAE methods. Figure 6b shows the ROC curves for short-pulse derived EDPTs (blue line), short-pulse DPOAE amplitudes for L_2_ = 55 dB SPL (orange line), and for two studies using conventional DPOAEs and TEOAEs (gray lines). The squares depict ROC curves extracted from a study by Gorga et al. [7] with 1,267 ears from 806 subjects, based on DPOAE amplitudes recorded with conventional stimulation at L_2_ = 55 dB SPL (L_1_ = 65 dB SPL) for f_2_ = 1.5 kHz (open squares) and f_2_ = 4 kHz (filled squares), respectively. Open circles correspond to TEOAE data from Gorga et al. [8] for a particularly suitable frequency range of one octave around 2 kHz. The comparison shows that a required sensitivity of 95% yields false-positive rates of only 5% for EDPTs and 15% for short-pulse DPOAE amplitudes, an accuracy that cannot even be closely achieved for conventional DPOAEs, even when limiting the analysis to a suitable frequency such as 4 kHz. The performance of TEOAEs is even worse than for conventional DPOAEs. The most convenient trade-off for TEOAEs would be a reduction of the required sensitivity to, say, 80%, which would still lead to a false-positive rate of 20%.

Analysis of DPOAEs in the time domain not only allows us to extract the nonlinear distortion component for achieving an objective diagnosis of higher accuracy, but also enables us to quantify the coherent reflection component [24], thus providing additional information about the functional integrity of the cochlear amplifier. In this context, Wagner et al. [23] reported that the prevalence of DPOAE fine structure significantly decreased with increasing hearing loss. Whereas the measurement of fine structure is time-consuming and technically demanding, a single, short-pulse DPOAE measurement can directly uncover the underlying components of the fine structure (e. g., Fig. 2b, c). However, until recently, pulsed DPOAE measurements were also prohibitively time-consuming. As an indicator of recent improvements, consider the acquisition time for DPOAE I/O functions with six primary-tone levels for eight audiometric frequencies between 1 and 8 kHz, based on our previous studies and data presented in this paper. While the acquisition time at introduction of the source-separation technique with pulsed primary tones was still about 2 h in 2009 [22], it was reduced to about 22 min in 2014 [26], and 8 min in the present study (Fig. 6) by reducing the pulse lengths and using optimized stimulus levels. For the near future, it is expected that, depending on the amount of cochlear amplifier damage, the acquisition time can be further reduced to somewhere between 2 and 5 min for these eight audiometric frequencies – by reducing the required number of DPOAEs per I/O function using adaptive stimulus paradigms.

## Conclusions for clinical practice

- Otoacoustic emissions reflect the functionality of the cochlear amplifier and are suitable for objective diagnosis of inner-ear impairment.
- DPOAEs comprise two components, which evolve at different places in the cochlea.
- Interference between these components hampers the accuracy of DPOAEs for evaluating the functionality of the cochlear amplifier.
- Short-pulse stimuli enable temporal separation of the DPOAE components.
- Compared with conventional DPOAE and TEOAE methods, the reliability for detecting impairment of the cochlear amplifier can be considerably increased by DPOAE component separation and optimal stimulus levels.
- With further reductions in acquisition time, the measurement procedure will be applicable in clinical routine in the near future.
